# Failure to recognise residual neuromuscular blockade in a patient with hemiparesis: a case-based report describing a limitation of quantitative neuromuscular monitoring

**DOI:** 10.1016/j.bjao.2026.100642

**Published:** 2026-08-14

**Authors:** Allart M. Venema, Tom J.P. Huberts, Carlotte G.A. van Dalen, Anthony R. Absalom, Marko M. Sahinovic

**Affiliations:** Department of Anaesthesiology, University Medical Center Groningen, Groningen, The Netherlands

**Keywords:** muscle denervation, neuromuscular blocking agents, neuromuscular monitoring, residual neuromuscular block, rocuronium, train-of-four monitoring

Editor

The use of non-depolarising neuromuscular blocking agents (n-NMBAs) necessitates intraoperative neuromuscular function monitoring, as undetected residual neuromuscular blockade increases the risk of postoperative complications. The use of intraoperative quantitative neuromuscular monitoring (QNMM), including calibration before administration of neuromuscular blocking agent, is therefore recommended by UK,^1^ USA^2^ and European guidelines.^3^

Although QNMM is generally considered reliable, it may produce misleading results in specific clinical scenarios such as hemiparesis or peripheral nerve injury. In these situations, functional adaptations at the neuromuscular junction (NMJ) alter the muscle’s response to peripheral nerve stimulation. Consequently, train-of-four (TOF)-ratio measurements may appear falsely reassuring despite clinically significant residual neuromuscular blockade. This limitation however, is not addressed in current guidelines.^1^, ^2^, ^3^ The aim of this letter is to describe a case in which failure to account for this limitation resulted in unrecognised residual neuromuscular blockade. This case highlights important clinical implications and underscores the need for formal recognition of this pitfall in future QNMM guidelines. The patient provided written informed consent for the publication of this letter.

During a busy night shift, we cared for an 81-yr-old male patient with pre-existing hemiparesis who underwent surgical drainage of a subdural haematoma under general anaesthesia. At the end of surgery, the TOF-ratio (measured on the paretic limb because of patient positioning and limited access) was 100%, indicating apparent complete recovery of neuromuscular function. After return of spontaneous breathing, accompanied by coughing, swallowing, and tongue protrusion on request, the trachea was extubated. However, the patient rapidly developed respiratory compromise. TOF-monitoring was then repeated on the non-paretic limb and showed residual neuromuscular blockade. Sugammadex administration led to rapid and complete recovery. A follow-up telephone call at 1 month confirmed continued recovery and a return to independent living.

## Discussion

“To err is human; to forgive, divine” (Alexander Pope, 1711) — a line later echoed by the Institute of Medicine’s 1999 report on patient safety.^4^ The complication we describe resulted from a combination of human and knowledge-related factors. Fatigue, the high cognitive workload associated with a busy night shift, and the emergency operation setting likely contributed to the event.^5^ However, the main issue was a knowledge-based error regarding the limitations of QNMM in patients with paretic or denervated muscles. Despite the deliberate use of QNMM to minimise the risk of residual blockade, the altered response of denervated muscle rendered the TOF ratio misleading.

The phenomenon of altered responses of denervated muscles to non-depolarising neuromuscular blocking agents (n-NMBAs) was first described in 1980 by D.H. Graham, who reported three cases involving pancuronium.^6^ Similar findings were later observed with other n-NMBAs, including rocuronium.^7^

These reports may reflect a broader systemic issue stemming from insufficient emphasis on the known pitfalls and limitations of neuromuscular monitoring. Errors arising from unrecognised clinical risk are inherently difficult to prevent and are likely to recur unless they are clearly specified in clinical guidelines.

The underlying pathophysiology has been well characterised. When neural input to muscle is lost by an upper or lower motor pathologic lesion, prolonged immobility, or critical illness, the NMJ undergoes a “denervation like” transformation. This process disrupts normal nerve-to-muscle signalling because of altered expression and distribution of acetylcholine receptors (AChRs) across the muscle membrane.^8^^,^^9^

Under normal conditions, mature muscle fibres express mature AChRs—pentamers composed of two α1, β1, δ, and ε subunits—that are tightly clustered at the NMJ to ensure efficient depolarisation and muscle contraction. In foetal muscle, before innervation, immature AChRs containing the γ subunit, and neuronal α7 AChRs are diffusely distributed across the muscle membrane. With innervation, these immature isoforms are repressed and replaced by mature receptors localised to the motor endplate.^9^

In denervation states, this pattern reverses: immature γ-containing AChRs and neuronal α7 AChRs are re-expressed and redistributed to extra-junctional regions of the muscle membrane. These receptors have altered electrophysiological properties for n-NMBAs. Pharmacodynamically these changes result in increased muscle excitability and resistance to neuromuscular blockade. Pharmacokinetically, widespread extra-junctional AChR expression acts as a diffusion sink for n-NMBAs, reducing drug availability at the NMJ.^9^ Together, these adaptations can produce falsely elevated TOF-ratios, as seen in our patient.

This problem is particularly relevant in patients undergoing neurosurgical procedures, where upper motor neuron lesions and reduced levels of consciousness are common. Given that annually, approximately 13.8 million patients worldwide suffer from a neurological disorder or injury that requires neurosurgery,^10^ the potential implications for patient safety are considerable. Greater awareness of this monitoring limitation is important to improve perioperative assessment and monitoring.

Current guidelines emphasise QNMM and pharmacological reversal when recovery is inadequate, but do not mandate routine reversal in all patients.^1^, ^2^, ^3^ Routine reversal may provide an additional safety margin, particularly when monitoring is unavailable, unreliable, or difficult to interpret. However, it may also be associated with adverse effects,^11^ increased cost, and practical limitations, including reduced options for short-term re-use of rocuronium.

**Fig 1:**
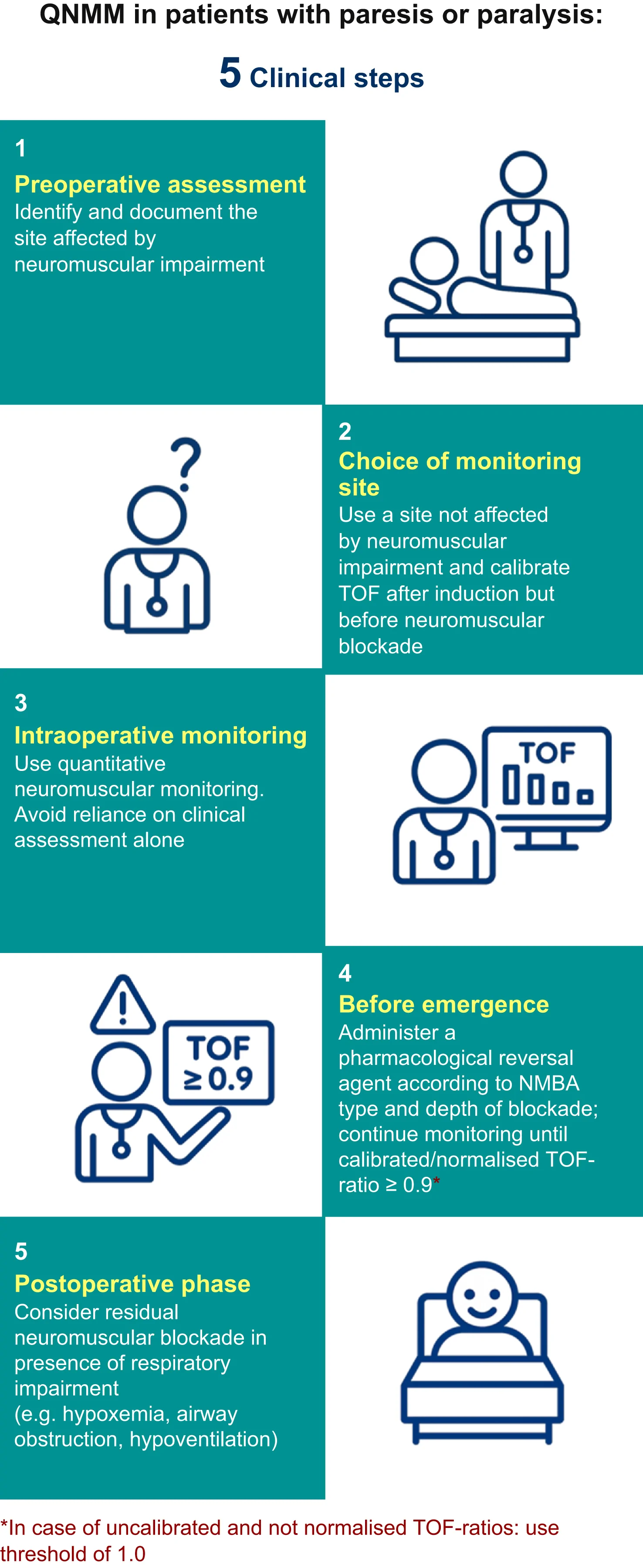
Infographic. Microsoft Copilot was used to assist in creating this infographic.

In conclusion, although QNMM is generally reliable, its limitations must be recognised. This case adds to existing reports highlighting the unreliability of TOF-monitoring in denervated muscles. We strongly recommend that current guidelines be amended to explicitly address these known limitations. To assist with this, we have developed practical suggestions for QNMM with particular emphasis on patients with paresis, paralysis, or denervation (Table 1 and Fig. 1).

**Table 1:** Practical considerations for quantitative neuromuscular monitoring in patients with paresis, paralysis, or denervation. AMG, acceleromyography; EMG, electromyography; TOF, train-of-four.

Phase	Recommendation	Details/notes
**Preoperative Assessment**	Identify site affected by neuromuscular impairment^9^	Assess for paresis, paralysis, neuropathy, or immobility affecting the limbs.
	Document affected and unaffected sides∗	Record the side and distribution of impairment in the anaesthetic record.
**Monitoring and site selection**	Use a non-affected site^9^	Use a site unaffected by neuromuscular impairment whenever feasible.
	If unavoidable^9^	Baseline TOF measurements may aid within-limb interpretation, but measurements may not reflect global recovery. Interpret with caution.
	Measure baseline TOF^1^, ^2^, ^3^	Use quantitative neuromuscular monitoring after induction of general anaesthesia but before administration of a neuromuscular blocking agent.
**Intraoperative monitoring**	Use quantitative techniques^2^	Use quantitative neuromuscular monitoring (e.g. AMG or EMG); avoid relying on clinical assessment alone.
**Before emergence**	Confirm recovery on unaffected site^2^^,^^3^	Confirm quantitative TOF: - If calibrated and normalised ensure TOF-ratio ≥ 0.9 before extubation. - If uncalibrated and not normalised ensure TOF-ratio= 1.0 before extubation.
	If adequate recovery cannot be confirmed^3^	Administer a pharmacological reversal agent according to the neuromuscular blocking agent and depth of blockade; continue monitoring until: - The calibrated and normalised TOF-ratiois ≥ 0.9 before extubation. - The uncalibrated and not normalised TOF-ratio is= 1.0 before extubation.
**Postoperative phase**	Maintain vigilance^2^^,^^12^	Consider residual neuromuscular blockade in the presence of respiratory impairment (e.g. hypoxaemia, airway obstruction, or hypoventilation).
	Document monitoring and recovery∗	Record the monitoring site and TOF ratio at extubation in the postoperative record.

∗ Pragmatic recommendation.

Greater awareness may help prevent similar failures to recognise residual neuromuscular blockade in patients with hemiparesis.

## Declarations of interest

The authors declare that they have no conflicts of interest.
